# Glycemic index and glycemic load of common fruit juices in Thailand

**DOI:** 10.1186/s41043-022-00284-z

**Published:** 2022-02-28

**Authors:** Chonnikant Visuthranukul, Pichet Sampatanukul, Suphab Aroonparkmongkol, Pathama Sirimongkol, Sirinuch Chomtho

**Affiliations:** 1 Pediatric Nutrition Research Unit, Division of Nutrition, Department of Pediatrics, Faculty of Medicine, Chulalongkorn University, Bangkok, 10330 Thailand; 2 Department of Pathology, Faculty of Medicine, Chulalongkorn University, Bangkok, 10330 Thailand; 3 Division of Endocrinology, Department of Pediatrics, King Chulalongkorn Memorial Hospital, The Thai Red Cross Society, Bangkok, 10330 Thailand; 4 Division of Nutrition, Department of Pediatrics, King Chulalongkorn Memorial Hospital, The Thai Red Cross Society, Bangkok, 10330 Thailand

**Keywords:** Glycemic index, Glycemic load, Postprandial glucose, Insulin, Fructose, Fruit juice

## Abstract

**Background:**

The glycemic index (GI) reflects body responses to different carbohydrate-rich foods. Generally, it cannot be simply predicted from the composition of the food but needs in vivo testing.

**Methods:**

Healthy adult volunteers with normal body mass index were recruited. Each volunteer was asked to participate in the study center twice in the first week to consume the reference glucose (50 g) and once a week thereafter to consume the study fruit juices in a random order. The study fruit juices were Florida orange juice, Tangerine orange juice, Blackcurrant mixed juice, and Veggie V9 orange carrot juice which were already available on the market. The serving size of each fruit juice was calculated to provide 50 g of glycemic carbohydrate. The fasting and subsequent venous blood samplings were obtained through the indwelling venous catheters at 0, 15, 30, 45, 60, 90, and 120 min after the test drink consumption and immediately sent for plasma glucose and insulin. GI and insulin indices were calculated from the incremental area under the curve of postprandial glucose of the test drink divided by the reference drink. Glycemic load (GL) was calculated from the GI multiplied by carbohydrate content in the serving size.

**Results:**

A total of 12 volunteers participated in the study. Plasma glucose and insulin peaked at 30 min after the drink was consumed, and then started to decline at 120 min. Tangerine orange juice had the lowest GI (34.1 ± 18.7) and GL (8.1 g). Veggie V9 had the highest GI (69.6 ± 43.3) but it was in the third GL rank (12.4 g). The insulin responses correlated well with the GI. Fructose to glucose ratio was inversely associated with GI and insulin responses for all study fruit juices. Fiber contents in the study juices did not correlate with glycemic and insulin indices.

**Conclusions:**

The GIs of fruit juices were varied but consistently showed a positive correlation with insulin indices. Fruit juices with low GI are a healthier choice for people with diabetes as well as individuals who want to stay healthy since it produces more subtle postprandial glucose and insulin responses.

## Background

The glycemic index (GI), an indicator ranking of carbohydrates according to their effects on the body’s postprandial glycemic response, was introduced more than 2 decades ago to facilitate glycemic control in patients with diabetes [1]. It was described as the percentage of incremental area under the 2-h blood glucose response curve of a test food divided by the corresponding area of a reference food containing the same amount of available carbohydrate (i.e. 50 grams (g) of glucose). The glycemic load (GL) was introduced more recently and is a product of GI multiplied by the amount of total available carbohydrates in a serving [2]. Since then, research has been extended to the role of a diet producing a low glycemic response (i.e. low GI and GL) in the prevention and management of metabolic syndrome [3], risk for type 2 diabetes [4], overweight and obesity [5], cardiovascular disease [6], and many other health problems, such as cancer [7] and age-related macular degeneration [8].

At present, we cannot predict GI value from specific type or amount of carbohydrate sources, dietary fiber or sugar content unless the response of physiological testing was done [1]. In 1997, a committee of experts was brought together by the Food and Agriculture Organization (FAO) of the United Nations and the World Health Organization (WHO) to review the available research that showed the importance of carbohydrates in human nutrition and health [9]. The committee endorsed the use of the GI method for classifying carbohydrate-rich foods and recommended that GI values of food should be used in conjunction with the information about the food composition to guide people in selecting healthy foods, especially those that contain carbohydrates. To promote good health, the committee advocated the consumption of a predominant carbohydrate diet (≥ 55% of energy from carbohydrate), with the bulk of carbohydrate-containing foods being rich in non-starch polysaccharides with a low GI (i.e. 55 or lower). For practical application, the GI is useful to rank foods by developing exchange lists of low GI foods. Moreover, specific local foods should be included in such lists where information is available because there is a wide variation in GI resulting from various intrinsic factors such as style of preparation (i.e. cooking, heat and moisture), physical form and inherent botanical difference of the food.

From the international table of GI and GL values 2008, low GI and GL foods contained fruit juices of various commercial brands from Australia and Canada (e.g. apple, orange and tomato juices). The GI and GL of these products varied [10]. Despite being one of the largest fruit suppliers in Asia, only a few fruits from Thailand have had their GI values identified [11, 12]. Therefore, the primary objective of this study was to quantify the GI and GL values of commercially available common fruit juices in Thailand to be included in the international GI and GL database. The secondary objective was to assess the association between GI and postprandial insulin responses as well as its correlation with fructose to glucose ratio and fiber content of different fruit juices.

## Methods

Healthy non-obese volunteers were recruited. People with underlying medical condition or were on medication known to affect the tolerance of glucose, had a first-degree family history of diabetes, was a heavy smoker and alcoholic drinker were excluded from the study. At baseline, all participants had normal fasting plasma glucose. Pregnant and lactating women were also excluded.

### Composition of the study drink

Detailed compositions of the tested juices are shown in Table 1. These study fruit juices have been available on the market at the time of this study (the Malee Brand, Samphran, Thailand). The available carbohydrate of the tested juices was calculated as the sum of free sugars (glucose, fructose, and sucrose) analyzed by High Performance Liquid Chromatography (HPLC) method under the standard of Association of Official Analytical Chemists (AOAC) 982.14 (NFI T997). There was no maltose or lactose presented; therefore, the result was not shown in the table. Dietary fiber was analyzed by an in-house method based on AOAC (2005) 985.29 (NFI 995). Both were done at the National Food Institute of Thailand (certified standard method by ISO IEC 17,025). Two samples per study juice were tested and the average was presented.

**Table 1:** Carbohydrate compositions and fiber content of the tested juices

Type of juice	Total sugar (g/dL)	Fructose (g/dL)	Glucose (g/dL)	Sucrose (g/dL)	Dietary fiber (g/dL)
100% Florida orange juice with orange pulp and sac	12.72	5.46	1.79	5.47	0.37
100%Tangerine orange juice with orange sac	11.89	6.95	1.28	3.66	0.53
100% Blackcurrant mixed strawberry and red grape juice	11.67	3.86	3.91	3.90	3.31
Veggie V9 –orange carrot, 40% mixed fruit juice, 60% mixed vegetable juice concentrate formula 2	8.91	3.06	3.12	2.73	1.78

The tested juices were served in the amount that provided 50 g of glycemic carbohydrate which was equivalent to 393 mL of Florida orange juice, 420 mL of Tangerine orange juice, 428 mL of Blackcurrant mixed, and 561 mL of Veggie V9.

### Postprandial study

The volunteers were asked to fast for 12 h after their usual evening meal, avoid exercise in the morning of the sample collection day, and abstain from alcohol the day before the tests were administered. At the clinic, a 24-h dietary recall was obtained. Next, the volunteer’s weight and height were recorded, and an intravenous catheter was inserted into a vein at the antecubital fossa. A fasting venous blood sample was taken, then, the participants were served either each tested fruit juice or reference drink within 5 min. Venous blood samples were drawn from the indwelling catheter at 15, 30, 45, 60, 90, and 120 min after the drink was consumed and immediately sent for laboratory analyses. The appointments for each volunteer were set to be at least one week apart. The postprandial study of 50 g glucose which was used as a reference drink was performed twice to consider the intra-individual variation in glycemic responses whereas the study for 4 different fruit juices was conducted once each in a random order. Therefore, each volunteer underwent 6 postprandial studies over the 6-week period.

### Laboratory analyses

Glucometer [Accu-check Performa test (Roche Diagnostic GmbH, Mannheim, Germany)] was also used to test small amount of the venous blood to provide point of care testing for each time points. Serum was immediately separated from the blood and stored at -20 °C until analysis. Plasma glucose concentrations were determined by enzymatic assay using the photometrically measured hexokinase method (Roche Diagnostic GmbH, Germany) at 0, 15, 30, 45, 60, 90, and 120 min. The insulin was measured by Immulite commercial test kit, a solid phase, two-site chemiluminescent immunometric assay (Siemens medical solutions diagnostics Ltd., Gwynedd, United Kingdom).

### Calculations and statistical analysis

The incremental area under the curve (IAUC) was calculated using the trapezoid method [9]. The GI was defined as the IAUC of the blood glucose response curve of a tested juice which was expressed as a percentage of the response to 50 g of glucose. All areas below the baseline were excluded from the calculation. The IAUC of the reference food (50 g of glucose) was calculated using the average of the 2 tests to avoid intra-subject variation of the glucose response to the food [13]. The GI was calculated based on each participant’s result and then reported as mean ± SD from 12 participants due to inter-individual variation in glucose and insulin responses to the food. The postprandial insulin responses were calculated in the same manner.

The correlations between glucose and insulin vs. carbohydrate composition as well as fiber content in the fruit juices was analysed by Spearman correlation using SPSS version 23.0 (SPSS Inc., Chicago, IL) P-value of < 0.05 was considered statistically significant.

The GL was calculated by multiplying the GI value to the amount of carbohydrate in a serving size (i.e. 200 mL) to take account for the realistic amount of carbohydrate consumed [14].

## Results

**Fig. 1:**
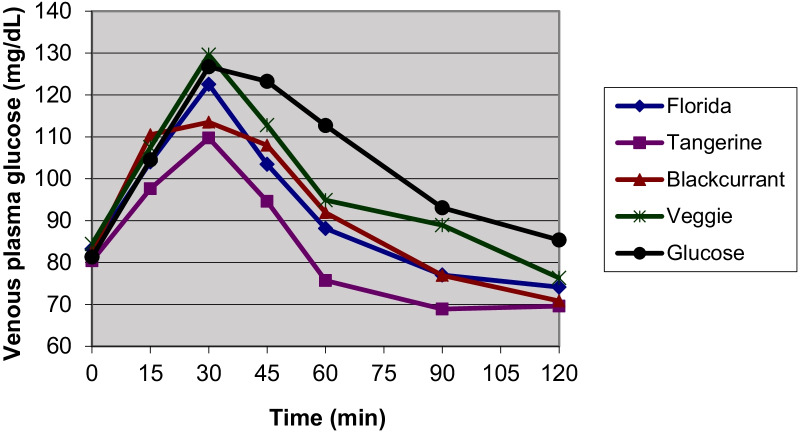
Mean fasting and postprandial venous plasma glucose responses in 10 participants over 120 min

**Fig. 2:**
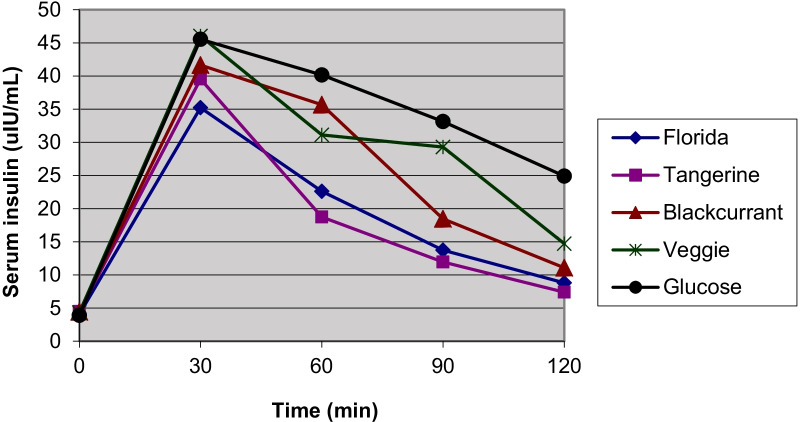
Mean fasting and postprandial serum insulin responses in 10 participants over 120 min

A total of 12 healthy volunteers, 7 women and 5 men, median aged 26.6 (IQR 5.8) years (range: 22–34 years) with normal body mass index (BMI) (21.5 (4.2) kg/m^2^ range: 19.2–24.7 kg/m^2^) participated in the study. Glucose solution produced a faster initial rise and a higher peak at 30 min in venous plasma glucose than most study juices except VeggieV9 (Fig. 1). The venous plasma glucose dropped below the fasting concentration at 120 min after the participants drank the study juices whereas after the consumption of glucose solution, it did not fall to baseline at 120 min. Plasma glucose at 45, 60, 90, and 120 min were significantly higher after the consumption of glucose compared to the study juices. The insulin responses showed a similar pattern with a highest peak at 30 min and a gradual decline over 120 min (Fig. 2). Nevertheless, the serum insulin did not go down to baseline at 120 min. Two participants showed abnormal glucose responses and were excluded from the analyses. The glycemic and insulin indices did not change significantly either with or without these two participants.

The GI, insulin indices, and carbohydrate composition of the tested juices are shown in Table 2. Tangerine orange juice has the lowest GI, followed by Florida orange juice, Blackcurrant mixed juice, and Veggie V9. According to the GI classification system [10], the first two were categorized as having low GI (≤ 55) and the latter two were categorized as having medium GI (56–69). Fructose to glucose ratio showed a significant negative correlation with GI and insulin indices by Spearman correlation Correlations between fructose to glucose ratio and plasma glucose were significant at 45 and 60 min whereas serum insulin was significant at 90 and 120 min. Sucrose to glucose ratio and fiber content did not show any significant correlation with glycemic and insulin indices.

**Table 2:** The glycemic and insulin indices and fructose/glucose and sucrose/glucose ratio of the tested juices

Tested juice	GI	CV (%)	Insulin index	CV (%)	Fructose: glucose ratio	Sucrose: glucose ratio
Florida orange juice	51.3 ± 25.9	50.47	53.4 ± 19.5	36.42	3.05	3.06
Tangerine orange juice	34.1 ± 18.7	54.66	51.0 ± 25.9	50.82	5.43	2.86
Blackcurrant mixed juice	63.0 ± 34.0	53.89	74.3 ± 29.0	38.99	0.99	1.00
Veggie V9–orange carrot	69.6 ± 43.3	62.11	86.4 ± 32.2	37.30	0.98	0.88

Data shows means ± SD

CV, coefficient of variation

From Table 3, the tangerine orange juice had the lowest GL (≤ 10) whereas the other three juices had medium GL (between 11 and 19) according to a GL classification system [14].

**Table 3:** The glycemic load of the tested juices calculated from a usual 200 mL serving size

Tested juice	GI	Serving size (mL)	Available carbohydrate (g)	GL (g)
Florida orange juice	51.3	200	25.4	13.0
Tangerine orange juice	34.1	200	23.8	8.1
Blackcurrant mixed juice	63.0	200	23.3	14.7
Veggie V9–orange carrot	69.6	200	17.8	12.4

GI: glycemic index; GL: glycemic load

## Discussion

In general, there are inter- and intra-individual differences in the glycemic responses to dietary intake [13].^.^ In our study, the measurement of GI values of the reference food (50 g of glucose) was performed twice and the glycemic responses of all foods were tested in the same participants under a standard condition to reduce the variations [9, 13, 15]. However, besides the effect of the foods that were tested, the preprandial glucose level and degree of insulin resistance of the participants may also affect the change of blood glucose concentration [15]. Brouns et al. recommended using subjects who have normal glucose tolerance for GI measurement because the variability of their glucose responses will be less than subjects with impaired glucose tolerance or diabetes [16]. Therefore, 2 volunteers with abnormal glucose tolerance were excluded for the final analysis.

To our knowledge, this is the first study that assessed the GI values of the fruit juices in Thailand. Previous studies have identified the GI values of some common Thai fruits [11, 12]. We postulated that the body glycemic response after eating a tangerine [12] and after drinking Tangerine orange juice would be different as demonstrated in our study. In the previous study, after the participants ate the tangerine (50 g of carbohydrate), the plasma glucose peaked at 60 min then dropped below baseline at 180 min [12]. However, when the participant drank the Tangerine orange juice, the venous plasma glucose peaked at 30 min and decreased below the baseline at 60 min. These results indicates that the glycemic response after drinking Tangerine orange juice seems to be faster than eating a tangerine resulting in its slightly higher GI of 34 compared to 30.

Both the glycemic and insulin indices in the Veggie V9 were the highest. Likewise, the GI value of the Tangerine orange juice and its insulin index were the lowest. The relationship between the glycemic response and insulin response in our study was similar to the previous reports [17, 18]. Wilson et al. reported that normal subjects and diabetic patients consuming different types of cranberry juice showed the positive correlation between insulin responses and glycemic responses. In addition, there was another study on glycemic and insulin responses of starchy foods [18]. This study demonstrated that starchy foods also had a correlation between GI and insulinemic index. Therefore, low GI beverage or diet could improve the insulin sensitivity because it gradually increase the plasma glucose and then slow down the secretion of insulin [19], may reduce insulin demand [20], and decrease β-cell dysfunction [21]. Consequently, diabetic patients may have a better blood glucose control which results in better health.

In general, the common compositions of the fruit juices were monosaccharide, disaccharides, and fiber contents. The GI values cannot be precisely predicted from the quantities or ratio of those compositions. Therefore, we studied the GI from four types of fruit juices by examining the glycemic responses in our participants to identify the actual GI in each fruit juice. However, there were many studies that tried to assess the correlation of the amount of fiber intake, the glycemic responses, and the occurrence of non-insulin-dependent diabetes mellitus (NIDDM) [2, 22–24]. The result was inconclusive. Some studies showed that patients with NIDDM who consumed high-fiber diets had a decrease in insulin demand [22]. Diabetic patients who ate starchy foods with soluble fibers had lower glycemic response than those who ingested starchy foods without soluble fibers [23]. In contrast, Jenkin et al. found that diabetic patients who ingested fiber-rich bread, such as whole meal bread, had the same blood glucose response compared to those who ingested white bread with low fiber contents [24]. Indeed, according to the international table of GI and GL values, the GI of these 2 types of bread are similarly high (74 and 75) despite the fact that the fiber content was much different [10]. Our study results also showed that there was no correlation between the fiber amounts and GI in the study fruit juices. Hence, we confirm that the amount of fiber contents in the fruit juices cannot predict GI value.

Since GI value of fructose is 20 which is lower than GI value of glucose [1], thus the postprandial blood glucose response may decrease when small amount of oral fructose is consumed [25]. Fructose causes the smallest increase in postprandial plasma glucose, postprandial glucose area, and serum insulin level when compared to other types of carbohydrate in healthy subjects and type 2 diabetic patients [26]. Therefore, we tested the hypothesis that fructose to glucose ratio could have a negative correlation with glycemic and insulin response. Tangerine orange juice had the highest fructose to glucose ratio while it had the lowest GI and GL in our study. The postprandial glucose is reduced after consuming fructose because it raises hepatic glucose uptake and decreases hepatic glucose output which results in lower postprandial glycemia [25]. The consumption of fructose is helpful in lowering serum glucose, insulin response, and glucosuria compared to dextrose and sucrose when given alone or as a component of a meal [27]. Moore et al. demonstrated that type 2 diabetic patients had an improvement in glucose tolerance when they consumed a small dose of fructose (7.5 g of fructose) which enhanced the net hepatic glucose uptake [28]. In contrast, Le et al. reported that healthy people consuming moderate amounts of fructose over 4 weeks had an increase in plasma triglyceride [29]. Furthermore, increased fructose consumption caused non-alcoholic fatty liver disease (NAFLD) in animals by increasing the fat mass and obesity [30], dyslipidemia [30], and induced insulin resistance as well as metabolic syndrome [31]. Moreover, human observational studies showed that there was a link between increased intake of fructose-containing beverages and accumulation of visceral adipose tissue [32] and intrahepatic lipids [33]. Hepatic lipid accumulation in the context of NAFLD is often associated with hepatic insulin resistance [34], and a crucial risk factor for type 2 diabetes [35]. There are many groups of researchers that have shown the benefits and risks of fructose ingestion as mentioned above; therefore, despite increasing the fructose to glucose ratio could lower GI of the fruit juice, we cannot recommend the fructose substitution in beverages.

The amount of carbohydrate intake is also an important factor in predicting body glycemic response. Since the GI may not definitely represent the amount of carbohydrate intake, thus the concept of GL has been used to show the amount of carbohydrate per usual serving size [14]. The GL is classified as low (≤ 10 g), medium (> 10- < 20 g), and high (≥ 20 g) [14]. Dietary GL might be useful to predict risk of type 2 diabetes [36]. According to our study results, we found that Florida orange juice had a low GI but medium GL due to a high carbohydrate per serving size. On the other hand, Veggie V9 had the highest GI in this study, but it was classified as medium GL because it had a low carbohydrate per serving size. These examples showed that the beverages low in GI do not necessarily had low GL. Therefore, consumers should consider both aspects simultaneously when choosing a healthier drink.

## Conclusions

Fruit juices, including the fruit juices tested in this study, are generally classified to have low to medium GI values. The glycemic indices of all fruit juices correlated well with in vivo insulin indices. Hence, fruit juices with low GI and GL could be advantageous compared to their higher GI and GL counterparts in terms of healthier insulin responses. However, GI or GL cannot be used as a sole indicator for ‘healthy food’ selection. Other composition such as protein, fat, fiber, and micronutrients should also be considered.

## Data Availability

The datasets used and/or analysed during the current study are available from the corresponding author on reasonable request.

## Abbreviations

AOAC: Association of Official Analytical Chemists
BMI: Body mass index
FAO: Food and Agriculture Organization
GI: Glycemic index
GL: Glycemic load
HPLC: High Performance Liquid Chromatography
IAUC: Incremental area under the curve
NAFLD: Non-alcoholic fatty liver disease
NIDDM: Non-insulin-dependent diabetes mellitus
WHO: World Health Organization

## References

[1] Jenkins DJ, Wolever TM, Taylor RH, Barker H, Fielden H, Baldwin JM (1981). Glycemic index of foods: a physiological basis for carbohydrate exchange. Am J Clin Nutr.

[2] Salmeron J, Manson JE, Stampfer MJ, Colditz GA, Wing AL, Willett WC (1997). Dietary fiber, glycemic load, and risk of non-insulin-dependent diabetes mellitus in women. JAMA.

[3] McKeown NM, Meigs JB, Liu S, Saltzman E, Wilson PW, Jacques PF (2004). Carbohydrate nutrition, insulin resistance, and the prevalence of the metabolic syndrome in the Framingham Offspring Cohort. Diabetes Care.

[4] Schulze MB, Liu S, Rimm EB, Manson JE, Willett WC, Hu FB (2004). Glycemic index, glycemic load, and dietary fiber intake and incidence of type 2 diabetes in younger and middle-aged women. Am J Clin Nutr.

[5] Murakami K, Sasaki S, Okubo H, Takahashi Y, Hosoi Y, Itabashi M (2007). Dietary fiber intake, dietary glycemic index and load, and body mass index: a cross-sectional study of 3931 Japanese women aged 18–20 years. Eur J Clin Nutr.

[6] Liu S, Willett WC, Stampfer MJ, Hu FB, Franz M, Sampson L (2000). A prospective study of dietary glycemic load, carbohydrate intake, and risk of coronary heart disease in US women. Am J Clin Nutr.

[7] Silvera SA, Rohan TE, Jain M, Terry PD, Howe GR, Miller AB (2005). Glycaemic index, glycaemic load and risk of endometrial cancer: a prospective cohort study. Public Health Nutr.

[8] Chiu CJ, Hubbard LD, Armstrong J, Rogers G, Jacques PF, Chylack LT (2006). Dietary glycemic index and carbohydrate in relation to early age-related macular degeneration. Am J Clin Nutr.

[9] Carbohydrates in human nutrition (1998). Report of a Joint FAO/WHO Expert Consultation. FAO Food Nutr Pap.

[10] Atkinson FS, Foster-Powell K, Brand-Miller JC (2008). International tables of glycemic index and glycemic load values: 2008. Diabetes Care.

[11] Somnuk S. Glycemic indexes of durian, mango, longan, pineapple, guava and dragon fruit and acute responses of plasma glucose, serum lipids and blood viscosity after ingestion different amounts of fruits in hyperlipidemic type 2 DM patients. Bangkok: Mahidol University; 2004. http://www.grad.mahidol.ac.th_grad_research_abstract_view.php_id=4336564&lang=en&fac=52&prg=5201M&gp=1.pdf. Accessed 22 Sep 2020.

[12] Chartchuathaicharoen P. Glycemic indexes of Tangerine, banana, papaya, rambutan, pamelo, and apple and acute responses of serum lipid and blood viscosity in DM type 2 patients. . Bangkok: Mahidol University; 2006. http://www.grad.mahidol.ac.th_grad_research_abstract_view.php_id=4536920&lang=en.pdf. Accessed 22 Sep 2020.

[13] Hatonen KA, Simila ME, Virtamo JR, Eriksson JG, Hannila ML, Sinkko HK (2006). Methodologic considerations in the measurement of glycemic index: glycemic response to rye bread, oatmeal porridge, and mashed potato. Am J Clin Nutr.

[14] Venn BJ, Wallace AJ, Monro JA, Perry T, Brown R, Frampton C (2006). The glycemic load estimated from the glycemic index does not differ greatly from that measured using a standard curve in healthy volunteers. J Nutr.

[15] Venn BJ, Green TJ (2007). Glycemic index and glycemic load: measurement issues and their effect on diet-disease relationships. Eur J Clin Nutr.

[16] Brouns F, Bjorck I, Frayn KN, Gibbs AL, Lang V, Slama G (2005). Glycaemic index methodology. Nutr Res Rev.

[17] Wilson T, Meyers SL, Singh AP, Limburg PJ, Vorsa N (2008). Favorable glycemic response of type 2 diabetics to low-calorie cranberry juice. J Food Sci.

[18] Lin MH, Wu MC, Lu S, Lin J (2010). Glycemic index, glycemic load and insulinemic index of Chinese starchy foods. World J Gastroenterol.

[19] Ludwig DS, Majzoub JA, Al-Zahrani A, Dallal GE, Blanco I, Roberts SB (1999). High glycemic index foods, overeating, and obesity. Pediatrics.

[20] Zawadzki JK, Bogardus C, Foley JE (1987). Insulin action in obese non-insulin-dependent diabetics and in their isolated adipocytes before and after weight loss. Diabetes.

[21] Radulian G, Rusu E, Dragomir A, Posea M (2009). Metabolic effects of low glycaemic index diets. Nutr J.

[22] Simpson HC, Simpson RW, Lousley S, Carter RD, Geekie M, Hockaday TD (1981). A high carbohydrate leguminous fibre diet improves all aspects of diabetic control. Lancet.

[23] Leclere CJ, Champ M, Boillot J, Guille G, Lecannu G, Molis C (1994). Role of viscous guar gums in lowering the glycemic response after a solid meal. Am J Clin Nutr.

[24] Jenkins DJ, Wolever TM, Jenkins AL, Lee R, Wong GS, Josse R (1983). Glycemic response to wheat products: reduced response to pasta but no effect of fiber. Diabetes Care.

[25] Wolf BW, Humphrey PM, Hadley CW, Maharry KS, Garleb KA, Firkins JL (2002). Supplemental fructose attenuates postprandial glycemia in Zucker fatty fa/fa rats. J Nutr.

[26] Bantle JP, Laine DC, Castle GW, Thomas JW, Hoogwerf BJ, Goetz FC (1983). Postprandial glucose and insulin responses to meals containing different carbohydrates in normal and diabetic subjects. N Engl J Med.

[27] Mayes PA (1993). Intermediary metabolism of fructose. Am J Clin Nutr.

[28] Moore MC, Davis SN, Mann SL, Cherrington AD (2001). Acute fructose administration improves oral glucose tolerance in adults with type 2 diabetes. Diabetes Care.

[29] Le KA, Tappy L (2006). Metabolic effects of fructose. Curr Opin Clin Nutr Metab Care.

[30] Softic S, Gupta MK, Wang GX, Fujisaka S, O'Neill BT, Rao TN (2017). Divergent effects of glucose and fructose on hepatic lipogenesis and insulin signaling. J Clin Invest.

[31] Balakumar M, Raji L, Prabhu D, Sathishkumar C, Prabu P, Mohan V (2016). High-fructose diet is as detrimental as high-fat diet in the induction of insulin resistance and diabetes mediated by hepatic/pancreatic endoplasmic reticulum (ER) stress. Mol Cell Biochem.

[32] Ma J, Sloan M, Fox CS, Hoffmann U, Smith CE, Saltzman E (2014). Sugar-sweetened beverage consumption is associated with abdominal fat partitioning in healthy adults. J Nutr.

[33] Ma J, Fox CS, Jacques PF, Speliotes EK, Hoffmann U, Smith CE (2015). Sugar-sweetened beverage, diet soda, and fatty liver disease in the Framingham Heart Study cohorts. J Hepatol.

[34] Kotronen A, Juurinen L, Tiikkainen M, Vehkavaara S, Yki-Järvinen H (2008). Increased liver fat, impaired insulin clearance, and hepatic and adipose tissue insulin resistance in type 2 diabetes. Gastroenterology.

[35] Anstee QM, Targher G, Day CP (2013). Progression of NAFLD to diabetes mellitus, cardiovascular disease or cirrhosis. Nat Rev Gastroenterol Hepatol.

[36] Willett W, Manson J, Liu S (2002). Glycemic index, glycemic load, and risk of type 2 diabetes. Am J Clin Nutr.

